# Multiple Comparisons With Overdispersed Multinomial Data: Methods, Properties and Application

**DOI:** 10.1002/pst.70073

**Published:** 2026-01-19

**Authors:** Sören Budig, Charlotte Vogel, Frank Schaarschmidt

**Affiliations:** ^1^ Department of Biostatistics Leibniz University Hannover Hannover Germany; ^2^ Independent Researcher Hannover Germany

**Keywords:** categorical data analysis, clustered data, multiple contrasts, quasi‐likelihood, zero counts

## Abstract

Overdispersion, a common issue in clustered multinomial data, can lead to biased standard errors and compromised statistical inference if not adequately addressed. This study describes a comprehensive procedure for constructing multiple comparisons of interest and applying multiplicity adjustments in the analysis of clustered, potentially overdispersed multinomial data. We investigate four quasi‐likelihood estimators and the Dirichlet‐multinomial model to account for overdispersion. Through a simulation study, we evaluate the performance of these methods under various scenarios, focusing on family‐wise error rate, statistical power and coverage probability. Our findings indicate that the Afroz quasi‐likelihood estimator is recommended when strict error control is required, whereas the Dirichlet‐multinomial model is preferable when high statistical power is desired, albeit with a slightly increased tolerance for false positives. Additionally, we address the challenge of zero‐count categories within groups, demonstrating that incorporating pseudo‐observations can effectively mitigate associated estimation difficulties. Practical applications to real datasets from toxicology and flow cytometry underscore the robustness and practical utility of these methods.

## 1 Introduction

Categorical data are collected in a wide range of research disciplines, including clinical trials, toxicology, genome sequencing, epidemiology and various fields within the life and social sciences. For example, in preclinical research, tissue sample damage in laboratory animals may be classified into various histopathological categories, while cell samples in clinical or preclinical trials can be classified into numerous categories through visual assessments (e.g., white blood cell differentials) or flow cytometry. Such data are derived by classifying the state of each experimental unit into one of several distinct categories. When the categories are hierarchically ordered, the data are classified as ordinal; otherwise, they are considered nominal. Our research focuses on the latter, commonly referred to as multinomial data in the literature.

A typical objective in studies involving multinomial data is to estimate and compare the probabilities associated with each category under different experimental or observational conditions. For instance, in a toxicological study, the state of an organism might be categorised as alive, malformed, or dead, with an interest in assessing the effect of different chemical doses on these outcomes. A multinomial model may be suitable for analysing such data. This model allows hypothesis testing, such as determining whether the ratio of dead to alive organisms is higher at increased doses compared to a control group. Additionally, the magnitude of this ratio may also be of interest. When multiple contrasts are of interest, multiple hypotheses may be tested, leading to the issue of multiple testing. For this inference problem, Schaarschmidt et al. [1] describe asymptotic methods for constructing simultaneous confidence intervals. They make use of the method described in Hothorn et al. [2], allowing for narrower intervals than those adjusted with Bonferroni, as it incorporates the correlation structure among the estimators.

A common challenge in this context is overdispersion, which refers to variability that exceeds the variance predicted by a basic multinomial model. Ignoring overdispersion can result in biased standard errors, overly narrow confidence intervals and potentially invalid conclusions [3]. The causes of overdispersion are many, ranging from complicated data collection procedures to inherent correlations between individual responses [4]. It is advisable to assume the presence of overdispersion until proven otherwise [5].

Several approaches exist to accommodate overdispersion in multinomial data. Quasi‐likelihood methods extend the variance by a multiplicative factor ϕ, which quantifies the degree of dispersion and can be estimated from the data [6]. Numerous estimators for ϕ have been proposed, offering robust and computationally efficient solutions [7, 8, 9, 10]. Additionally, extended distributions, such as the Dirichlet‐multinomial (DM) and other compound distributions, inherently account for overdispersion [11, 12, 13, 14]. Other approaches involve the utilisation of mixed models to address overdispersion through the incorporation of random effects [15, 16, 17], or the application of generalised estimating equations [18].

This paper extends the work of Schaarschmidt et al. [1] by examining various methods that account for overdispersion in multinomial data and incorporating these adjustments into multiple comparison procedures to derive appropriately adjusted p‐values and simultaneous confidence intervals. Specifically, we compare four quasi‐likelihood estimators and the application of a compound distribution, the DM distribution, in terms of family‐wise error rate (FWER), power and coverage probability.

Furthermore, a frequently encountered issue is that certain categories within groups predominantly or exclusively contain zeros, which can result in extreme estimates, inflated standard errors and difficulties in rejecting null hypotheses. To address this, we explore the heuristic of adding one pseudo‐observation to zero‐count categories within groups.

In the following sections of this paper, we will revisit the definitions of the parameters of interest, describe the methods for addressing overdispersion and outline the computation of multiplicity adjustments. The methods will be evaluated and compared through a simulation study. Finally, we will demonstrate the application of these methodologies using three real data examples from toxicology and experimental biology.

## 2 Methods

### 2.1 Data Structure

Consider a completely randomised design with G different treatment groups, indexed by g=1,…,G. Each group g contains a fixed number of clusters, $B_{g}$, predetermined by the experimental design. The dependent variable is nominal and represents the count of experimental units in each group that can be assigned to one of several categories, indexed by c=1,…,C. Each experimental unit belongs to a cluster $b_{g}=1,\ldots ,B_{g}$, which may result in non‐independent observations within clusters. Let $m_{\mathrm{gb}}$ be the total number of experimental units within cluster $b_{g}$ and $m_{g}$ be the total number of experimental units in group g. It is assumed that there are no secondary factors or covariates influencing the experimental outcomes.

A common assumption for this data type is that the count vector $y_{\mathrm{gb}}=\left(y_{\mathrm{gb}1},y_{\mathrm{gb}2},\ldots ,y_{\mathrm{gbC}}\right)$ for the C categories provided by cluster b within group g follows a multinomial distribution:

$$y_{\mathrm{gb}}∼\text{multinomial}\left(m_{\mathrm{gb}},\pi_{g}\right),$$

where $\pi_{g}=\left(\pi_{g1},\pi_{g2},\ldots ,\pi_{\mathrm{gC}}\right)$ is a row vector consisting of the probabilities that an experimental unit in group g falls into category c. The variance of a single count is given by $V\left(y_{\mathrm{gbc}}\right)=m_{\mathrm{gb}}\pi_{\mathrm{gc}}\left(1-\pi_{\mathrm{gc}}\right)$. Note that in many biological applications, the variance of an observed count may differ from the variance assumed in the multinomial distribution.

### 2.2 Parameters of Interest

Analysing such data often involves comparing groups based on the relative change in odds. On a logarithmic scale, this comparison is expressed as differences in log odds, also known as log odds ratios. For instance, to compare the first two treatment groups regarding the first two categories, the log odds ratio would be $\log\left(\left(\pi_{22}/\pi_{21}\right)/\left(\pi_{12}/\pi_{11}\right)\right)$. Depending on the scientific question, different comparisons or parameters may be of interest. First, according to Schaarschmidt et al. [1], a set of log odds of interest within a given group g can be defined using

(1) $$\delta_{g}=A\log\left(\pi_{g}^{T}\right),$$

where A is an I×C matrix containing all comparisons of interest. Each row is a separate contrast vector that defines which two categories are to be compared (odds). Subsequent comparisons of these odds between groups result in log odds ratios. If the between‐group comparisons remain consistent across all odds, the parameter vector θ is expressed as:

(2) $$\theta =\left(B⊗A\right)\left(\begin{array}{c} \log\;\pi_{1}^{T}\\ \log\;\pi_{2}^{T}\\ \vdots \\ \log\;\pi_{G}^{T} \end{array}\right),$$

where B denotes the contrast matrix for between‐group comparisons of dimension J×G. The column vector θ comprises log odds ratios arranged by between‐group comparisons (j=1,…,J) and within these by category odds (i=1,…,I). By stacking the column vectors $\delta_{g}$ into a single column vector $\delta ={\left(\delta_{1}^{T},\delta_{2}^{T},\ldots ,\delta_{G}^{T}\right)}^{T}$, θ can also be calculated using

$$\theta =\left(B⊗I_{I}\right)\delta ,$$

where $I_{I}$ represents the identity matrix of dimension I×I [1]. In both matrices A and B, irrelevant rows can be removed and various contrast matrices of interest can be constructed, such as those analogous to Tukey [19] or Dunnett [20]. Additionally, contrast matrices for comparisons with the grand mean or for testing trends may be applicable. It is important to note that the number of contrasts increases rapidly as the number of categories and groups increases and the more hypotheses are tested, the stronger the multiplicity adjustment becomes.

### 2.3 Overdispersion

When analysing multinomial data, it is common to observe greater variation in the data than expected under the multinomial model, known as overdispersion. Overdispersion may be caused by several factors, including unobserved heterogeneity, additional sources of experimental variability, or inherent model flaws. It can also result from correlations among experimental units within clusters that are not accounted for in the basic multinomial framework. Recognising and addressing overdispersion is critical, as ignoring it can lead to erroneous conclusions and flawed statistical inferences. It can affect the accuracy of parameter estimates and lead to biased standard errors, resulting in incorrect hypothesis testing.

Several approaches can be used to account for potential overdispersion in multinomial data. These include quasi‐likelihood approaches [21] and alternative or extended distributions, such as the Dirichlet‐multinomial (DM) distribution [14]. In this study, we evaluate and compare four quasi‐likelihood estimators alongside the DM distribution as a parametric method to accommodate overdispersion.

Within the quasi‐likelihood framework, a dispersion parameter (ϕ) is estimated to quantify the excess variance observed in the data and scale the variance accordingly. Assuming that the random variable $y_{\mathrm{gb}}$ follows a multinomial distribution, the mean and covariance are given as:

$$E\left(y_{\mathrm{gb}}\right)=m_{\mathrm{gb}}\pi_{g},$$

$$\mathrm{Cov}\left(y_{\mathrm{gb}}\right)=\phi \Sigma_{y\mathrm{gb}},$$

where ϕ>0 and $\Sigma_{y\mathrm{gb}}$ denotes the multinomial variance–covariance matrix, expressed as $\Sigma_{y\mathrm{gb}}=m_{\mathrm{gb}}\left\{\text{Diag}\left(\pi_{g}^{T}\right)-\pi_{g}^{T}\pi_{g}\right\}$ [5].

In addition to the quasi‐likelihood approach, the DM distribution provides a parametric alternative for dealing with overdispersion in multinomial count data. This distribution extends the multinomial distribution by assuming that the vector of proportions $\pi_{\mathrm{gb}}$ follows a Dirichlet distribution with a vector of shape parameters $\alpha_{g}=\left(\alpha_{g1},\alpha_{g2},\ldots ,\alpha_{\mathrm{gC}}\right)$. Hence, we model $\pi_{g}∼\text{Dirichlet}\left(\alpha_{g}\right)$ and $y_{\mathrm{gb}}∼\text{multinomial}\left(m_{\mathrm{gb}},\pi_{\mathrm{gb}}\right)$ [11]. This prior distribution introduces additional flexibility in the variance structure of the model and the covariance includes a corrective term to account for potential additional variance:

$$\mathrm{Cov}\left(y_{\mathrm{gb}}\right)=\left(\frac{m_{\mathrm{gb}}+\alpha_{g.}}{1+\alpha_{g.}}\right)\Sigma_{y\mathrm{gb}},$$

where $\alpha_{g.}=\sum_{c}\alpha_{\mathrm{gc}}$ [22].

To simulate multinomial data with specified levels of overdispersion, we assume equal cluster sizes ($m_{\mathrm{gb}}=m$) and homogeneous dispersion across groups ($\alpha_{g.}=\alpha$). A dispersion parameter, analogous to those in quasi‐likelihood methods, can be defined as:

(3) $$\phi_{\mathrm{DM}}=\frac{m+\alpha }{1+\alpha },$$

which illustrates that when α becomes large, $\phi_{\mathrm{DM}}$ approaches 1 and the DM distribution reverts to a multinomial distribution. By rearranging Equation (3), the scale parameter α corresponding to the desired level of overdispersion and cluster size can be computed as:

$$\alpha =\frac{\phi_{\mathrm{DM}}-m}{1-\phi }.$$

For each cluster $b_{g}$, random probability vectors $\pi_{\mathrm{gb}}$ for each cluster b in group g can be sampled from a Dirichlet distribution using the parameters $\alpha_{g}=\alpha \pi_{g}$. These probability vectors are then used to generate observation vectors $y_{\mathrm{gb}}$ from the multinomial distribution with probability $\pi_{g}$ and equal cluster size m. When the cluster sizes $m_{\mathrm{gb}}$ are uniform across experimental units gb, the simulated data conform to both the quasi‐multinomial assumption of McCullagh and Nelder [5], assuming a common dispersion factor ϕ and the DM assumption. Notably, the methods described in this study remain applicable to datasets with unequal cluster sizes.

### 2.4 Estimation

To fit the plain multinomial model and estimate its parameters, we use the vglm function from the VGAM package [23]. The argument family = multinomial can be specified within the function to assume a multinomial distribution for the data. In this case, maximum likelihood estimation is used to estimate the parameters. This function directly estimates the log odds with a chosen baseline category for a chosen reference group and the differences between the log odds of the remaining groups and the reference group. Throughout the simulations, we use the first category c=1 as the baseline category and the first group g=1 as the reference group. The estimated parameter vector can be described as $\beta ={\left(\log\frac{\pi_{12}}{\pi_{11}},\log\frac{\pi_{13}}{\pi_{11}},\ldots ,\log\frac{\pi_{1C}}{\pi_{11}},\log\frac{\pi_{22}/\pi_{21}}{\pi_{12}/\pi_{11}},\ldots ,\log\frac{\pi_{\mathrm{GC}}/\pi_{G1}}{\pi_{1C}/\pi_{11}}\right)}^{T}$. To calculate the parameter vector θ with the comparisons of interest we can use $\theta =\left(B^{*}⊗A^{*}\right)\beta$, where $A_{\left(I\times C-1\right)}^{*}$ is the same matrix as $A_{\left(I\times C\right)}$ but omits the first column and $B_{\left(J\times G\right)}^{*}$ is the same matrix as $B_{\left(J\times G\right)}$ but the first column is set to 0.

Several quasi‐likelihood estimators exist to estimate ϕ and for our simulation study, we compare four estimators. First, the estimator proposed by Wedderburn [6] is based on Pearson's $\chi^{2}$‐statistics, normalised by the degrees of freedom of the full model:

$${\hat{\phi }}_{P}=\chi^{2}/\left(N-n-P\right),$$

where df=N−n−P are the residual degrees of freedom, where $n=\sum_{g=1}^{G}B_{g}$ is the total number of clusters, N=nC and P denotes the number of non‐redundant parameters [5]. If no other covariates or factors are present in the model, P can be computed using $P=G\left(C-1\right)$. Pearson's lack‐of‐fit statistic is calculated as $\chi^{2}=\sum_{g=1}^{G}\sum_{b_{g}=1}^{B_{g}}\sum_{c=1}^{C}{\left(y_{\mathrm{gbc}}-m_{\mathrm{gb}}{\hat{\pi }}_{\mathrm{gc}}\right)}^{2}/m_{\mathrm{gb}}{\hat{\pi }}_{\mathrm{gc}}$, where $m_{\mathrm{gb}}{\hat{\pi }}_{\mathrm{gc}}$ are the estimated expected counts from the fitted model [24].

The second estimator, based on deviance statistics, is computed as:

$${\hat{\phi }}_{D}=D/\left(N-n-P\right),$$

where $D=2\sum_{g=1}^{G}\sum_{b_{g}=1}^{B_{g}}\sum_{c=1}^{C}y_{\mathrm{gbc}}\log\left(y_{\mathrm{gbc}}/m_{\mathrm{gb}}{\hat{\pi }}_{\mathrm{gc}}\right)$ [25].

Two additional estimators, incorporating degrees of freedom adjustments, were described by Afroz et al. [10]. The first, based on the extensions by Deng and Paul [9] from Farrington [7], is computed as:

$${\hat{\phi }}_{F}={\hat{\phi }}_{P}-\left(N-n\right)\bar{s}/\left(N-n-P\right),$$

and 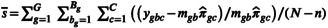. The other estimator is based on the work of Fletcher [8] and extended by Afroz et al. [10] to the application of multinomial data and is computed as:

$${\hat{\phi }}_{A}={\hat{\phi }}_{P}/\left(1+\bar{s}\right).$$

As stated by Afroz et al. [10], sparse data in multinomial analyses may lead to inaccuracies when testing lack‐of‐fit using $\chi^{2}$ and D statistics, as their distributions deviate significantly from the assumed $\chi_{N-n-P}^{2}$ distribution. Consequently, estimators ${\hat{\phi }}_{P}$ and ${\hat{\phi }}_{D}$ might underperform under conditions of sparse data, with the latter being potentially worse. According to Afroz et al. [10], the estimator ${\hat{\phi }}_{A}$ is preferable due to asymptotic theory and simulation results.

The DM model introduces an additional layer of complexity by inherently accounting for overdispersion in the data. Within the vglm function, the argument family = dirmultinomial is used to assume a DM distribution for the data. Maximum likelihood estimation is performed using a Fisher scoring algorithm, which may be computationally intensive, particularly for larger sample sizes [13, 26]. Therefore, an alternative implementation of the DM distribution is also considered, using the MGLMreg function from the MGLM package [27]. This implementation utilises an iteratively reweighted Poisson regression method for maximum likelihood estimation. However, an alternative parametrisation is used in which the parameters $\eta_{g}=\left(\eta_{g1},\eta_{g2},\ldots ,\eta_{\mathrm{gC}}\right)$ are estimated. How these parameters are related to the shape parameters of the Dirichlet multinomial distribution can be found in Kim et al. [27]. The parameters of interest are then computed by $\theta =\left(B⊗A\right)\eta$, where the first C columns of the resulting matrix of $\left(B⊗A\right)$ are set to 0 and $\eta ={\left(\eta_{1},\eta_{2},\ldots ,\eta_{G}\right)}^{T}$. These parameters can also be converted into the parameter vector β, analogous to the one estimated by the vglm function, using $\beta =\left(I_{G}⊗A_{C-1\times C}\right)\eta$, where $I_{G}$ is the G×G identity matrix and $A_{C-1\times C}$ is a Dunnett‐type contrast matrix for the categories.

Alternative algorithms for the DM model that improve accuracy and runtime have been presented by Yu and Shaw [28], Languasco and Migliardi [29] and Baddar et al. [30]. However, as these algorithms have not yet been implemented in user‐friendly software, we have opted to use the VGAM and MGLM implementations for simulation.

### 2.5 Adjustment for Multiple Comparisons

As described in Section 2.2, comparisons between groups for the log odds of categories can be conducted. These comparisons entail testing whether an element of the parameter vector θ deviates from a specified value, commonly set to zero. The hypotheses can be formulated as follows:

$$H_{0k}:\theta_{k}=0\;\mathrm{vs}.\;H_{\mathrm{Ak}}:\theta_{k}\neq 0,$$

where ${\hat{\theta }}_{k}$ is the *k*th element of $\hat{\theta }$. We use the methodology of Hothorn et al. [2], which is applicable for p‐value adjustment in hypothesis testing and for generating simultaneous confidence intervals for θ. The use of this method for constructing simultaneous confidence intervals for parameters of a multinomial model was outlined by Schaarschmidt et al. [1] and is briefly recalled here. The estimator of the parameter vector $\theta =\left(B⊗I_{I}\right)\delta$ asymptotically follows a multivariate normal distribution with mean θ and variance covariance matrix V [24]. The variance–covariance matrix V of the log odds differences is expressed as:

$$V=\left(B⊗I_{I}\right)\Sigma {\left(B⊗I_{I}\right)}^{T},$$

where Σ is the variance–covariance matrix of the estimator $\hat{\delta }$, configured as a block diagonal matrix containing the individual covariance matrices of the independent estimators ${\hat{\delta }}_{g}$ on the diagonal:

$$\Sigma =\left(\begin{array}{cccc} \phi \Sigma_{1} & 0 & \cdots & 0\\ 0 & \phi \Sigma_{2} & \cdots & 0\\ \vdots & \vdots & & \vdots \\ 0 & 0 & \cdots & \phi \Sigma_{G} \end{array}\right).$$

With each ${\hat{\delta }}_{g}$ having the asymptotic covariance matrix

$$\Sigma_{g}=m_{g}^{-1}\left(A\text{Diag}{\left(\pi_{g}\right)}^{-1}A^{T}-A11^{T}A^{T}\right),$$

where $\text{Diag}{\left(\pi_{g}\right)}^{-1}$ is the inverse of the diagonal matrix with the $\pi_{g}$ elements and 1 is an C×1 vector of 1 elements [24]. The estimators $\hat{\theta }$, $\hat{\Sigma }$, ${\hat{\Sigma }}_{g}$ and $\hat{V}$ of θ, Σ, $\Sigma_{g}$ and V can be derived using the sample proportion ${\hat{\pi }}_{g}$ and the respective $\hat{\phi }\in \left\{{\hat{\phi }}_{P},{\hat{\phi }}_{D},{\hat{\phi }}_{F},{\hat{\phi }}_{A}\right\}$ in place of $\pi_{g}$ and ϕ. For hypothesis testing, we use the test statistic:

$$t_{k}=\frac{{\hat{\theta }}_{k}}{\sqrt{{\hat{v}}_{k}}},$$

where ${\hat{v}}_{k}$ is the estimated variance of the respective log odds ratio, that is, the *k*th diagonal element of the variance–covariance matrix $\hat{V}$. To simultaneously assess the hypotheses of interest, a critical value for comparison with our test statistics must be determined. We opt for a critical value $q_{1-\alpha ,K,\mathrm{df},\hat{R}}$ as the two‐sided quantile of a multivariate *t*‐distribution [2] with correlation $\hat{R}$, where $\hat{R}$ denotes the standardised variance–covariance matrix $\hat{V}$ [31]. We employ the multivariate *t*‐distribution instead of the multivariate normal distribution for the distribution of the test statistics under the null hypothesis, given that the true variance–covariance matrix is unknown and we may encounter small sample sizes [32]. If $q_{1-\alpha ,K,\mathrm{df},\hat{R}}$ are the elements of a multivariate random vector $Z_{K,0,\mathrm{df},\hat{R}}$ with expectation 0 and correlation matrix $\hat{R}$, then the critical value $q_{1-\alpha ,K,\mathrm{df},\hat{R}}$ is chosen such that $P\left(\max_{k=1,\ldots ,K}\left(q_{k}\right)\leq q_{1-\alpha ,K,\mathrm{df},\hat{R}}\right)=1-\alpha$. If an absolute test statistic exceeds the critical value, that is, $|t_{k}|\;>q_{1-\alpha ,K,\mathrm{df},\hat{R}}$, we can reject the corresponding null hypothesis. Subsequently, asymptotically adjusted p‐values for each of the hypotheses of interest can be calculated by $p_{k}=P\left(\max_{k=1,\ldots ,K}\left(q_{k}\right)>|t_{k}\right)$. Alternatively, two‐sided simultaneous confidence intervals can be constructed as follows:

$$\exp\left[{\hat{\theta }}_{k}\pm q_{1-\alpha ,K,\mathrm{df},\hat{R}}\sqrt{{\hat{v}}_{k}}\right],\;k=1,\ldots ,K.$$

## 3 Simulation Study

### 3.1 Simulation Setup

A comprehensive simulation study was carried out to investigate the properties of the full procedure described. The four quasi‐likelihood methods and the two DM models were compared. Throughout the simulations, we maintained C=3 categories and G=4 treatment groups. We considered 60 different scenarios in which the true proportions of the categories were either constant or varied across the treatment groups. Among these, 21 scenarios had no treatment effect, meaning the proportions of the categories were equal among groups, while 39 scenarios exhibited treatment effects, indicated by different log odds between the treatment groups. A table of these scenarios is available in the Appendix A. In each scenario and setting, the first category was consistently used as the baseline category for logits, with comparisons made either versus the first treatment group as the control (using Dunnett‐type contrasts between categories and between groups) or pairwise between all treatment groups (using Dunnett‐type contrasts between categories and Tukey‐type contrasts between groups). We selected $B_{g}\in \left\{5,10,20,50\right\}$ as the number of clusters per treatment group, with each cluster having an equal size, set at $m_{\mathrm{gb}}\in \left\{10,20,50,100\right\}$. The overdispersion was set to $\phi \in \left\{1.01,1.5,2,5,8\right\}$.

In total, 1000 independent datasets were generated for each combination of parameter settings and scenarios, using both sets of odds ratios, drawn from the DM distribution. For each dataset, both the multinomial model and two variants of the DM model (VGAM (DM_VGAM) and MGLM (DM_MGLM) implementations) were fitted. Multiplicity adjustments were applied to the models. For the multinomial model, the four different dispersion parameters were used independently. Thus, seven methods were systematically applied to each dataset, including the plain multinomial model and the two DM models. We evaluated the FWER and the statistical power for each method. The FWER is the probability of incorrectly rejecting at least one null hypothesis in a series of tests. Power was defined as the proportion of correctly rejected false null hypotheses. In addition, simultaneous 95% confidence intervals were computed and the coverage probability, which is defined as the proportion of simulated datasets for which the intervals of all contrasts contained the corresponding true parameter value, was calculated. Furthermore, we estimated the bias, its standard error and the mean squared error for the log odds and dispersion parameters.

A specific issue addressed in our simulations was the occurrence of extreme standard errors resulting from the observation of zeros in all clusters of a given group and category, preventing the rejection of null hypotheses involving such groups. To mitigate this, we performed an additional simulation in which a count of 1 was added to a random cluster of such group and category, an approach similar to that proposed by, for example, Gart [33] or Goodman [34]. In this additional simulation, we used the same scenarios and log odds ratios, but we applied a slightly smaller parameter space of $b_{g}\in \left\{5,10,20\right\}$ and $m_{\mathrm{gb}}\in \left\{10,20\right\}$ to reduce computation time. The dispersion remained as previously specified and the same quantities as before were estimated.

The main simulation study presented here relies on two assumptions: data generation from the DM distribution and homogeneous dispersion (ϕ) across all treatment groups. Further we always used equal cluster sizes ($m_{\mathrm{gb}}=m$) within each experiment. To assess the robustness of the evaluated methods when these assumptions are violated or when variable cluster sizes are used, additional simulation studies were conducted. These further investigations explored scenarios involving variable cluster sizes drawn from a Poisson distribution, heterogeneous dispersion parameters across treatment groups and data generated from a logistic normal multinomial (LNM) distribution to examine model misspecification, including a scenario combining LNM generation with variable cluster sizes. The setup and results of these supplementary simulations are detailed in the Supporting Information (Section S1).

All simulations were executed using R software version 4.3.3. For sampling from the Dirichlet distribution, the MCMCpack package [35] was employed. Model fitting for the multinomial model and the DM model (via the vglm function) was performed using the VGAM package [23] and an alternative implementation of the DM model was carried out using the MGLMreg function from the MGLM package [27]. For multiplicity adjustments, the multcomp package [2] was employed. To maintain conciseness, we present only the results for FWER and power. Full results—including estimates of coverage probability, bias, standard error and mean squared error—along with detailed descriptions of the simulation study and the corresponding R code, are available in the accompanying dataset on the GitHub repository (https://github.com/sbudig/multnom_multcomp).

### 3.2 Simulation Results

**FIGURE 1:**
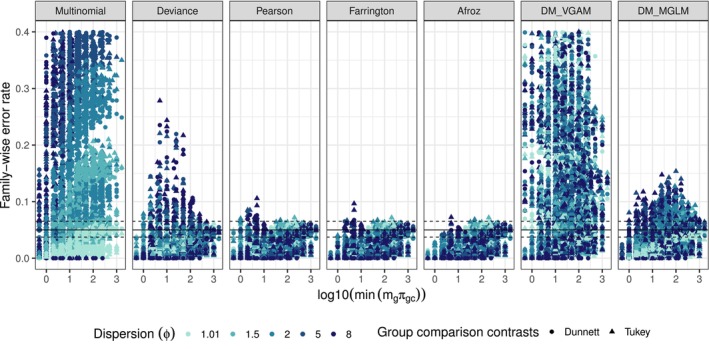
Simulated FWER of the six methods for all scenarios and settings. The colour indicates the size of the dispersion ϕ and the shape distinguishes the two sets of log odds (Tukey and Dunnett). The nominal level of 0.05 is represented by the solid horizontal line and the dashed lines represent the standard error of the simulation. The limit of the *y*‐axis was set to 0.4.

Figure 1 presents the FWER stratified by method across all scenarios, parameter settings and two sets of log odds ratios. On the x‐axis, $\log10\left(\min\left(m_{g}\pi_{\mathrm{gc}}\right)\right)$ represents the log10‐transformed minimum expected counts across all clusters and categories within groups. To facilitate comparison among methods, the *y*‐axis limit was set at 0.4.

Overall, the plain multinomial model shows an increase in FWER with higher dispersion (ϕ), reaching values up to 0.95 in scenarios with a dispersion of 8. Incorporating overdispersion using quasi‐likelihood approaches yields different behaviours among estimators. The deviance estimator appears somewhat liberal for large ϕ and small $\min\left(m_{g}\pi_{\mathrm{gc}}\right)$ but becomes conservative as $\min\left(m_{g}\pi_{\mathrm{gc}}\right)$ increases, frequently falling below the nominal level. The Pearson, Afroz and Farrington estimators consistently control the FWER, converging toward the nominal level as $\min\left(m_{g}\pi_{\mathrm{gc}}\right)$ increases. However, these methods also tend toward conservatism across various scenarios. The distinctions among these three estimators are minor, although at lower $\min\left(m_{g}\pi_{\mathrm{gc}}\right)$ values, the Pearson estimator often remains closest to the nominal level. However, it can occasionally appear liberal in scenarios with higher dispersion. Conversely, the Farrington and Afroz estimators are generally more conservative at lower $\min\left(m_{g}\pi_{\mathrm{gc}}\right)$ values, with the Farrington estimator being slightly more liberal at higher dispersion settings, whereas the Afroz estimator consistently controls the FWER across all examined scenarios. Regarding the DM implementations, the VGAM version (DM_VGAM) appears insensitive to dispersion but frequently fails to adequately control the FWER, with values reaching as high as 0.94 in some cases. A closer analysis indicates overly liberal FWER behaviour in scenarios characterised by low probabilities of occurrence in the reference category. However, once these probabilities exceed approximately 0.45 in the reference category, the VGAM implementation either controls the FWER or tends to be conservative. In contrast, the MGLM implementation (DM_MGLM) consistently performs better, reliably controlling the FWER when dispersion is around 1. At dispersions above 1, there are occasional scenarios where the FWER increases up to 0.15, though generally, a convergence towards nominal levels occurs with increasing $\min\left(m_{g}\pi_{\mathrm{gc}}\right)$. Differences between the two sets of log odds (Tukey vs. Dunnett) are minimal. Although Tukey comparisons typically exhibit slightly higher FWER, these differences are negligible; therefore, further comparisons between these sets are not pursued in subsequent figures.

**FIGURE 2:**
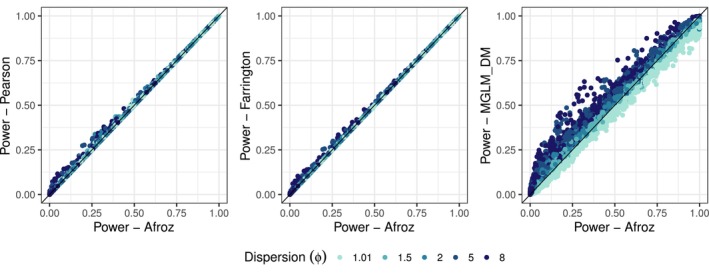
Comparison of the Pearson, Afroz and Farrington estimators and the DM model (MGLM implementation) in terms of simulated power for all scenarios and settings. The colour indicates the size of the dispersion ϕ.

Figure 2 compares the statistical power among the Pearson, Afroz and Farrington quasi‐likelihood estimators and the DM MGLM implementation. The power results broadly align with earlier observations concerning FWER. The Pearson and Farrington estimators consistently yield similar or higher power compared to the Afroz estimator. Compared with the DM model, the Afroz estimator has higher power for dispersions around one, while the DM model consistently has higher power for dispersions above one.

**FIGURE 3:**
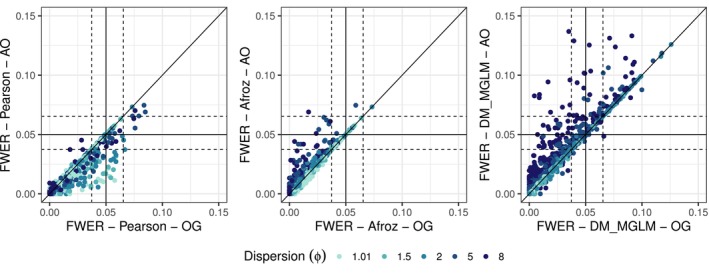
Comparison of the FWER of the modified (AO: Adding 1 observation to random cluster) to the original dataset (OG) for the three methods Pearson, Afroz and DM (MGLM implementation) for all scenarios and settings. The result with the original dataset is shown on the *x*‐axis and the result with the modified dataset is shown on the *y*‐axis. The colour indicates the size of the dispersion ϕ. The nominal level of 0.05 is represented by the solid horizontal and vertical lines and the dashed lines represent the standard error of the simulation.

An additional simulation investigated the effect of adding a single observation to a randomly selected cluster within a category and group initially lacking observations. Notably, this scenario occurs infrequently or not at all in some simulation settings, resulting in minimal observable differences. Figure 3 compares the FWER for the Pearson, Afroz estimators and the DM model (MGLM implementation) between the original dataset (OG) and the modified dataset (AO). The Pearson estimator tends to be more liberal with the original dataset, particularly at lower dispersion. Conversely, the Afroz estimator displays marginally less conservative FWER behaviour when using the modified dataset, especially at higher dispersion. For the DM model, the dataset modification generally results in a notably higher FWER, particularly in scenarios with increased dispersion, reaching values up to threefold compared to the original dataset.

**FIGURE 4:**
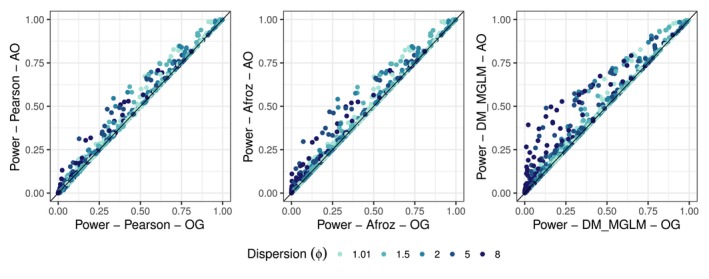
Comparison of the power of the modified (AO: Adding 1 observation to random cluster) to the original dataset (OG) for the three methods Pearson, Afroz and DM (MGLM implementation) for all scenarios and settings. The result with the original dataset is shown on the *x*‐axis and the result with the modified dataset is shown on the *y*‐axis. The colour indicates the size of the dispersion ϕ.

Figure 4 compares power between the original (OG) and modified datasets (AO) for the Pearson, Afroz estimators and DM model (MGLM). The two quasi‐likelihood estimators demonstrate comparable behaviour, with the modified dataset providing power gains up to approximately 25% in some settings. The DM model similarly benefits from the modification, though it often exhibits greater power improvements, especially in higher dispersion settings, achieving gains up to around 35%.

Supplementary simulations further assessed the robustness of the methods (Supporting Information, Section S1). Introducing variable cluster sizes (drawn from a Poisson distribution) while maintaining DM data generation had minimal impact on the FWER control compared to the fixed cluster size scenarios presented above. However, simulating heterogeneous dispersion across groups led to inflated FWER (up to 0.1) for the quasi‐likelihood methods, particularly when dispersion levels varied widely, while the DM (MGLM) model showed the same performance as in the simulations above. When the data generating process was changed to the logistic normal multinomial (LNM) distribution, all methods exhibited substantially liberal FWER (up to 0.2 for quasi‐likelihood, 0.38 for DM), indicating sensitivity to deviations from the assumed distribution, including misspecification of the assumed mean–variance relation. Adding variable cluster sizes to the LNM generation slightly worsened these results in specific cases, adding to the effect of the initial model misspecification.

To further investigate computational cost, we performed a scalability simulation (Supporting Information, Section S2). The results show that the model fitting step for the quasi‐likelihood approach is consistently fast, whereas the DM models (both VGAM and MGLM) can become computationally intensive in scenarios with many groups, categories and large cluster sizes. For all methods, the primary computational bottleneck is the multiple comparison adjustment. The time for this step increases substantially with the number of comparisons, with scenarios involving ≈950 comparisons taking nearly an hour on one CPU core (5.7 GHz). We also note a practical constraint, as the pmvt function used for the adjustment does not support more than 1000 comparisons.

## 4 Real Data Application

### 4.1 Example 1: Differential Blood Count

**FIGURE 5:**
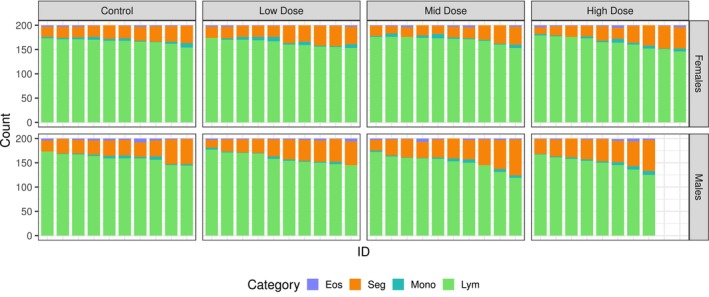
Bar chart for the counts of white blood cells (Eosinophils [Eos], Segmented Neutrophils [Seg], Monocytes [Mono] and Lymphocytes [Lym]) from the differential blood count example. The individual bars represent the counts from the same rat. The rows indicate the sex of the rat and the columns the dose of a toxin.

The first example involves a toxicological study of white blood cell counts in rats. The raw data were obtained from Hothorn [36] and were previously analysed in a similar manner by Schaarschmidt et al. [1], though without accounting for overdispersion. The study compared four treatment groups: one control group and three groups exposed to varying doses of a toxin (low, mid and high). Rats of both sexes were randomly assigned to the dose groups. Each group consisted of 10 male and 10 female rats, except for the high dose group, which consisted of only eight male rats. For each rat, 200 leukocytes were counted and classified into six categories: Eosinophils, Basophils, Neutrophilic Bands, Segmented Neutrophils, Monocytes and Lymphocytes. The total count per rat was predetermined by the experimental design and the categories were unordered. Figure 5 presents the leukocyte counts for each category. Each bar represents an individual rat, with row panels indicating sex and column panels indicating toxin dose. Basophil and neutrophil classifications were absent from the leukocyte counts and are therefore excluded from the graph and subsequent analysis. Despite identical experimental conditions, variations in leukocyte counts among rats are evident. This suggests the potential presence of overdispersion.

The primary research question was whether the proportions of the different white blood cell classifications varied significantly between the control group and the toxin dose levels in both male and female rats. To address this, we calculated all pairwise odds (I=6) among the C=4 categories. To utilise these odds for comparisons between the low, mid and high dose levels with the control, stratified by sex, we combined the two factors, group and sex, into a new factor, resulting in J=6 distinct between‐group comparisons. The corresponding matrices A and B are:

$$A_{\left(I\times C\right)}=\left(\begin{array}{cccc} -1 & 1 & 0 & 0\\ -1 & 0 & 1 & 0\\ -1 & 0 & 0 & 1\\ 0 & -1 & 1 & 0\\ 0 & -1 & 0 & 1\\ 0 & 0 & -1 & 1 \end{array}\right),\;B_{\left(J\times G\right)}=\left(\begin{array}{cccccccc} -1 & 1 & 0 & 0 & 0 & 0 & 0 & 0\\ -1 & 0 & 1 & 0 & 0 & 0 & 0 & 0\\ -1 & 0 & 0 & 1 & 0 & 0 & 0 & 0\\ 0 & 0 & 0 & 0 & -1 & 1 & 0 & 0\\ 0 & 0 & 0 & 0 & -1 & 0 & 1 & 0\\ 0 & 0 & 0 & 0 & -1 & 0 & 0 & 1 \end{array}\right).$$

**FIGURE 6:**
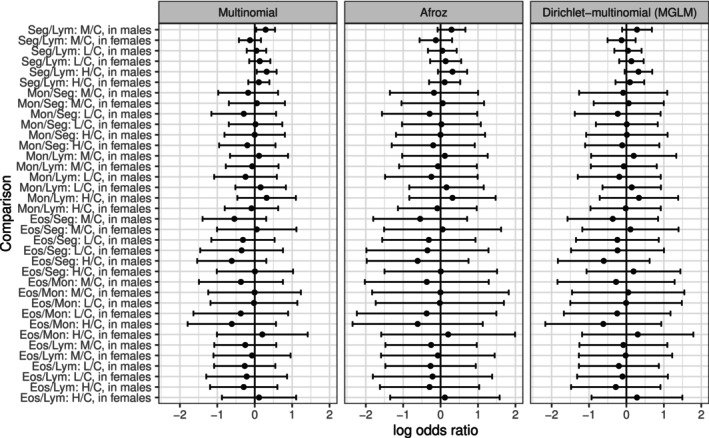
Estimated simultaneous 95% confidence intervals for the 36 log odds ratios described in the differential blood count example.

Table 1 presents the estimates for the log odds ratios, along with the corresponding standard errors and p‐values, for a subset of the total 36 comparisons. These comparisons were carried out using a multinomial model, once without adjustment for overdispersion and once using the Afroz dispersion estimator ${\hat{\phi }}_{A}=2.17$. After adjusting for multiple comparisons using the Afroz method, the odds comparisons in M/C and H/C for males are no longer significant, as they were when using the plain multinomial model. Figure 6 displays the simultaneous confidence intervals for the log odds ratios, analogous to those presented in Schaarschmidt et al. [1]. Here, the DM model was also used (MGLM implementation). It is evident that the confidence intervals derived from the multinomial model are the smallest. In contrast, the intervals obtained using the Afroz method are the largest overall, while the DM model generally yields slightly smaller intervals. Notably, no significant odds ratios were observed in either method that account for overdispersion.

**TABLE 1:** Differential blood count example results of selected subset: Estimated log odds ratios, standard errors and p‐values for the comparison of each dose group with the control using the plain multinomial model and the Afroz method.

Linear hypotheses	$\hat{\theta }$	$\mathrm{SE}{\left(\hat{\theta }\right)}_{\text{mult}}$	$\mathrm{SE}{\left(\hat{\theta }\right)}_{\text{Afroz}}$	$p_{\text{mult}}$	$p_{\text{Afroz}}$
Seg/Lym: L/C, in females	0.136	0.090	0.133	0.923	0.998
Seg/Lym: M/C, in females	−0.127	0.095	0.139	0.969	1.000
Seg/Lym: H/C, in females	0.110	0.091	0.134	0.988	1.000
Seg/Lym: L/C, in males	0.047	0.084	0.123	1.000	1.000
Seg/Lym: M/C, in males	0.291	0.081	0.119	0.010	0.283
Seg/Lym: H/C, in males	0.319	0.085	0.125	0.006	0.227

### 4.2 Example 2: Developmental Toxicity

**FIGURE 7:**
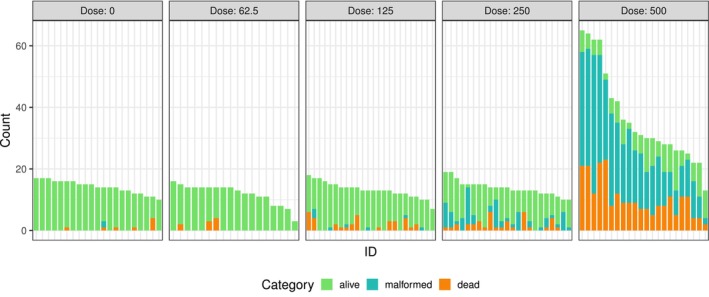
Bar chart for counts of alive, malformed or dead foetuses of mice exposed to DYME (0, 62.5, 125, 250 or 500 mg/kg/day) from the developmental toxicity example. Each bar represents the number of foetuses from a single mouse, with bars per dose level sorted by total foetus count.

The dataset originates from a study conducted by the National Toxicology Program [37] on maternal toxicity in timed‐pregnant CD‐1 mice following exposure to diethylene glycol dimethyl ether (DYME), a widely used organic solvent classified among trichothecene mycotoxins known for its high developmental toxicity. The research aimed to explore the effects of DYME on foetal development and offspring survival. Pregnant mice were randomly assigned to receive different doses of DYME (0, 62.5, 125, 250, or 500 mg/kg/day) administered orally from gestational days 6 to 15. The mice were sacrificed on gestational day 17 and the offspring were categorised as alive, malformed, or dead. This dataset was previously analysed by Hothorn [36] using two quasi‐binomial models and the R package multcomp [2] to model their joint distribution. The dataset is included in the SiTuR package, which accompanies the referenced book. Figure 7 displays a bar chart of the number of foetuses per mouse for each category across the different dose levels. The chart shows variation in the number of mice per dose level and suggests that higher doses may affect the number of foetuses delivered. In addition, the variability between clusters in the number of dead or malformed foetuses within each dose group indicates potential overdispersion in the data. Notably, no malformed foetuses were recorded at the 62.5 mg/kg dose level.

To test whether increasing dosages significantly affect the proportion of dead or malformed foetuses relative to those alive, we applied the methods described above to the data. Given the absence of malformed foetuses at the 62.5 mg/kg dose level, we added a single malformed observation to the mouse with the highest offspring count at this dose to avoid extremely large standard errors and estimates. Both a multinomial and two DM models (VGAM and MGLM implementations) were fitted to estimate the occurrence probabilities of each category per dose level, using alive as the baseline category. A Dunnett‐type contrast matrix was then used to compare the log odds between each dose group and the control. The multinomial model provided the basis for calculating the four dispersion parameters described, which were then multiplied with the variance–covariance matrix to account for possible overdispersion. The estimated dispersion parameters were ${\hat{\phi }}_{\text{pearson}}=2.416$, ${\hat{\phi }}_{\text{afroz}}=2.461$, ${\hat{\phi }}_{\text{farrington}}=2.435$ and ${\hat{\phi }}_{\text{deviance}}=2.017$. Finally, the function glht from the multcomp package was used to adjust for multiple comparisons for the multinomial model (with each of the four dispersion parameters) and the DM models (see the above Github link for more details in the corresponding R‐code). The results, including estimates, standard errors, p‐values and the lower and upper limits of the 95% simultaneous confidence intervals for odds ratios using the Afroz method, are shown in Table 2. Significant increases in the odds of malformed relative to alive foetuses were observed at the 250 and 500 mg/kg dose levels compared with the control, with ratios increased by a factor of [2.42, 906.98] at the 250 mg/kg dose and by a factor of [22.19, 7847.52] at the 500 mg/kg dose. Similarly, the odds of dead relative to alive foetuses were significantly increased at the 250 and 500 mg/kg doses by factors [1.22, 32.40] and [12.13, 259.36], respectively. Adding one malformed observation at the 62.5 mg/kg dose level reduced the estimated standard error for the malformed/alive odds from 1184.26 to 1.93 (on the log scale).

**TABLE 2:** Developmental toxicity example results: Estimated log odds ratios, standard errors, p‐values, back‐transformed odds ratios and their lower and upper limits of the simultaneous 95% confidence intervals for the comparison of each dose group with the control using the Afroz method.

Linear hypotheses	$\hat{\theta }$	$\mathrm{SE}\left(\hat{\theta }\right)$	p‐value	$\exp\left(\hat{\theta }\right)$	2.5%	97.5%
malformed/alive: 62.5–0	−0.35	1.93	1.00	0.70	0.00	107.75
malformed/alive: 125–0	1.44	1.25	0.73	4.20	0.16	109.33
malformed/alive: 250–0	3.85	1.13	0.00	46.87	2.42	906.98
malformed/alive: 500–0	6.03	1.12	0.00	417.31	22.19	7847.52
dead/alive: 62.5–0	0.46	0.78	0.98	1.58	0.21	11.99
dead/alive: 125–0	1.53	0.63	0.08	4.60	0.89	23.77
dead/alive: 250–0	1.84	0.63	0.02	6.28	1.22	32.40
dead/alive: 500–0	4.03	0.59	0.00	56.08	12.13	259.36

### 4.3 Example 3: Flow Cytometry Counts of Four Cell Types

**FIGURE 8:**
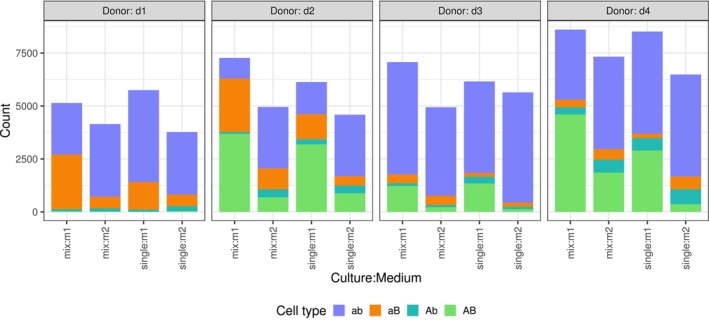
Bar chart for the counts of cell types (ab, Ab, AB, aB) from the flow cytometer example. The individual bars represent the counts of the same culture medium mix for each donor, with the column panels indicating the donor.

In this example, flow cytometry was utilised to analyse the cellular properties across different treatment groups. The experiment involved four donors (d1, d2, d3, d4), each providing a sample of human stem cells. Each sample was divided into four treatment groups, determined by the combination of two factors: the type of culture (single or mix) and the type of medium used (m1, m2). Data collection involved classifying 10,000 cells per donor and treatment group using flow cytometry. In some cases, the available material was insufficient to reach 10,000 cells. Additionally, a portion of the cells could not be classified due to clumping and some cells were dead. The remaining living cells were categorised based on two markers, each with two states (aA, bB), resulting in four cell types: ab, Ab, AB and aB. Figure 8 displays a bar plot derived from the data. To demonstrate the ability to answer a wide range of questions, we assume that we are interested in the comparison of the odds between the two media types and between the two culture types. In addition, we want to test whether the medium type influences the effect of the culture type or vice versa, that is, the interaction between these two factors. We have therefore created a new factor with four levels that combines the levels of culture and medium type (the order of the levels is the same as the *x*‐axis labels in the Figure 8). We have chosen the ab cell type as the baseline category. The corresponding contrast matrices A and B are:

$$A_{\left(I\times C\right)}=\left(\begin{array}{cccc} -1 & 1 & 0 & 0\\ -1 & 0 & 1 & 0\\ -1 & 0 & 0 & 1 \end{array}\right),\;B_{\left(J\times G\right)}=\left(\begin{array}{cccc} -0.5 & 0.5 & -0.5 & 0.5\\ 0.5 & 0.5 & -0.5 & -0.5\\ 1 & -1 & -1 & 1 \end{array}\right).$$

The first row of the $B_{\left(J\times G\right)}$ matrix contains the weights used for the comparison of the difference between m1 and m2 across both single and mix cultures. The second row tests the difference between single and mix cultures across both m1 and m2 media. The third row tests the interaction between medium and culture type, by looking at how the effect of switching from m1 to m2 changes when single instead of mix culture is used. The donor was treated as an additive block factor. More details can be found in the corresponding R code at the GitHub link above.

Table 3 presents the estimates of the log odds ratios, their standard errors, adjusted p‐values and the lower and upper limits of the simultaneous 95% confidence intervals for the Afroz method. Generally, there is very high overall overdispersion in the data and the resulting dispersion parameter estimates for the four methods are: ${\hat{\phi }}_{P}=150.21$, ${\hat{\phi }}_{A}=123.76$, ${\hat{\phi }}_{F}=149.83$, ${\hat{\phi }}_{D}=139.32$. If an analysis were to ignore this overdispersion and instead assume a multinomial distribution with ϕ=1, all the comparisons of interest would become highly significant. When considering the comparisons based on the Afroz estimator, the odds aB/ab and AB/ab are significantly smaller in m2 compared to m1. No significant interaction between medium and culture type was observed.

**TABLE 3:** Flow cytometry example results: Estimated log odds ratios, standard errors, p‐values, back‐transformed odds ratios and their lower and upper limits of the simultaneous 95% confidence intervals for the parameters described in the experiment using the Afroz method.

Linear hypotheses	$\hat{\theta }$	$\mathrm{SE}\left(\hat{\theta }\right)$	p‐value	$\exp\left(\hat{\theta }\right)$	2.5%	97.5%
Ab/ab: m2‐m1	0.15	0.36	1.00	1.17	0.40	3.40
aB/ab: m2‐m1	−0.87	0.25	0.01	0.42	0.20	0.87
AB/ab: m2‐m1	−1.79	0.24	0.00	0.17	0.08	0.34
Ab/ab: mix‐single	−0.23	0.36	1.00	0.79	0.27	2.30
aB/ab: mix‐single	0.72	0.24	0.05	2.06	1.00	4.26
AB/ab: mix‐single	0.63	0.23	0.10	1.87	0.93	3.75
Ab/ab: interaction	−0.31	0.72	1.00	0.73	0.09	6.21
aB/ab: interaction	0.66	0.49	0.81	1.93	0.45	8.22
AB/ab: interaction	−0.28	0.47	1.00	0.76	0.19	3.05

## 5 Discussion

This paper highlights the importance of addressing potential overdispersion and adjusting for multiple comparisons when analysing clustered multinomial data. We adopted the methodology of Schaarschmidt et al. [1], which facilitates the computation of adjusted p‐values and simultaneous confidence intervals for contrast sets defining multiple odds ratios. We extended their approach by incorporating potential overdispersion using one of four dispersion parameters within a quasi‐likelihood framework or by using a DM model.

Our main simulation results, which are based on scenarios motivated primarily by toxicological and pharmaceutical applications, reveal that the four dispersion estimators exhibit similar properties. With the exception of the deviance estimator, all effectively control the FWER across most of the investigated scenarios and settings. The performance of the DM model, however, depends on the implementation used. Specifically, the VGAM implementation becomes excessively liberal when the probability of occurrence in the reference category is low but adequately controls the FWER when this probability exceeds approximately 0.45. Given this limitation, the VGAM implementation is impractical for general use. Conversely, the MGLM implementation demonstrates better performance, although it exhibits slightly liberal behaviour in some scenarios with dispersion greater than one.

Our power simulations indicate that high power is achievable with all dispersion estimators, even in the presence of high overdispersion. The Pearson estimator generally exhibits the highest power, followed by the Farrington estimator. The Afroz estimator, while slightly less powerful, provides the smallest bias in estimating the dispersion parameter (ϕ), consistent with the findings of Afroz et al. [10]. Thus, the Afroz dispersion estimator is recommended for analyses where strict error control is required, whereas the DM model (MGLM) is suggested for scenarios where increased power can justify slightly higher false positive rates.

We also investigated the impact of violated assumptions and variable cluster sizes in supplementary simulations (Supporting Information, Section S1). When cluster sizes were allowed to vary according to a Poisson distribution, the performance of both quasi‐likelihood and DM (MGLM) methods remained largely consistent with the main findings, suggesting reasonable robustness to this variability. Introducing heterogeneous dispersion across groups, however, revealed potential limitations. While the DM (MGLM) model showed similar performance to the main simulations, the quasi‐likelihood methods (Pearson, Farrington and Afroz estimators), estimating a single global dispersion parameter, became liberal (FWER up to 0.11) when true dispersion levels differed substantially between groups. Furthermore, simulating data from a logistic normal multinomial (LNM) distribution to assess model misspecification led to considerably inflated FWER (up to 0.2 for quasi‐likelihood methods, 0.4 for DM model), especially with higher variance and covariance in the LNM's latent structure. While this misspecification was the primary driver of inflated error rates, combining LNM generation with variable cluster sizes led to a further slight increase in the FWER in specific scenarios. These supplementary results highlight the sensitivity of the methods to significant dispersion heterogeneity and misspecification of distributional assumption. This suggests that alternative methods should be employed when the model's assumptions appear to be violated, as revealed by residual diagnostics, for example.

Estimating group‐specific dispersion parameters and incorporating them into the variance–covariance matrix is possible and could yield more robust inferences. Further studies are needed to explore this potential. Alternatively, using mixed models with random cluster effects could be explored as an additional method for addressing overdispersion [15, 16, 17].

An improvement to the quasi‐likelihood approach could involve the use of bootstrap methods to estimate ϕ [10]. However, computational time may become prohibitive as model complexity increases.

Currently, the procedure for computing multiple contrasts of interest and applying a multiplicity adjustment when dealing with overdispersed multinomial data is not directly implemented in R. Therefore, we provide custom code for the example analyses and our simulation study on GitHub (https://github.com/sbudig/multnom_multcomp). The code provided for the examples can easily be adapted to other datasets.

Throughout this paper, we have focused on multiple comparison procedures that control the family‐wise error rate (FWER). This choice was motivated by applications in confirmatory research, such as toxicology studies, where the consequences of a single false positive finding are critical and strong error control is necessary. In other, more exploratory contexts with a large number of comparisons, controlling the false discovery rate could offer a more powerful alternative by controlling the expected proportion of false discoveries. The unadjusted p‐values from our models, computed prior to any FWER adjustment, could serve as input for standard FDR procedures. However, a primary goal of our approach is the construction of simultaneous confidence intervals, which provide inference on effect magnitude [38]. Standard FDR‐controlling procedures are designed for hypothesis testing and do not typically yield simultaneous confidence intervals.

In this paper, we have addressed excess variability in multinomial data by employing models that account for overdispersion at the cluster level. This framework is particularly well‐suited to data from biological and toxicological studies, where the cluster is a natural experimental unit (e.g., a litter of animals) and the primary source of extra‐multinomial variation is the biological variability between these units. An alternative and complementary perspective, which is prominent in the social and behavioural sciences, attributes excess variation to unobserved heterogeneity at the individual level. The Heterogeneous Multinomial Logit Model (HMLM), as described by Tutz [39], provides a framework for this viewpoint. Rather than modelling a single overdispersion parameter, the HMLM is a population‐averaged approach that models individual‐level variation as a function of observed covariates. It uses a location‐scale framework where a scaling term, itself a function of covariates like age or gender, modifies the effect strength of the main predictors. This allows researchers to investigate how choice uncertainty or variability is influenced by specific, measurable characteristics. This individual‐level approach is relevant for disciplines where the objective is to understand the complex and varied decision‐making of individual subjects. While the development and evaluation of methods for multiple comparisons within the HMLM framework would exceed the scope of this article, it does represent a basis for future research.

In both our simulations and real data analyses, zero counts within category groups often resulted in extreme estimators and inflated standard errors, leading to large confidence intervals and difficulty in rejecting the null hypothesis. Similar problems, known as the Hauck‐Donner effect in binomial data, are typically addressed by adding pseudo‐observations (e.g., 0.5 or 1) to cells of contingency tables. Analogously, we investigated adding a count of 1 to a random cluster within a zero count group. We acknowledge that this pragmatic approach has ad‐hoc elements. More principled alternatives have been proposed in related contexts. For example, Firth's penalised likelihood [40], often used for binary data, provides finite estimates in cases of data separation by penalising the likelihood function. A fully Bayesian model using weakly informative priors would also naturally stabilise estimates without ad‐hoc adjustments [41]. While these methods are theoretically superior, their application and validation for simultaneous inference within the overdispersed multinomial framework are complex and beyond the scope of this paper. Our proposed adjustment, in contrast, is computationally simple and, as our simulations show, controlled the FWER across most scenarios and settings with the Afroz, Pearson and Farrington estimators, sometimes increasing power by reducing extreme standard errors. The DM model also showed a power advantage in some settings using this approach, though the nominal FWER was sometimes exceeded.

Our simulations were restricted to scenarios with three categories and four groups and thus the methods' performance in scenarios involving more categories remains uncertain. Increasing the number of categories may worsen issues of sparse counts, warranting further research.

Overall, all presented methods are capable of handling more complex models with additional substructures or covariates. However, defining a suitable contrast matrix for multiple comparisons in such models can quickly become challenging.

## Funding

The authors have nothing to report.

## Conflicts of Interest

The authors declare no conflicts of interest.

## Supporting information


**Data S1:** pst70073‐sup‐0001‐supinfo.pdf.

## Data Availability

The data that support the findings of this study are openly available in multnom_multcomp at https://github.com/sbudig/multnom_multcomp.

## Appendix A Multinomial Proportions Used in Simulation Study

See Table A1.

**TABLE A1:** Proportions ($\pi_{\mathrm{gc}}$) for each category (c) and group (g) across the different scenarios used in the simulation study.

	c=1	c=2	c=3	c=1	c=2	c=3	c=1	c=2	c=3	c=1	c=2	c=3	c=1	c=2	c=3	c=1	c=2	c=3
g=1	0.33	0.33	0.33	0.50	0.30	0.20	0.50	0.40	0.10	0.50	0.45	0.05	0.46	0.45	0.09	0.80	0.10	0.10
g=2	0.33	0.33	0.33	0.50	0.30	0.20	0.50	0.40	0.10	0.50	0.45	0.05	0.46	0.45	0.09	0.80	0.10	0.10
g=3	0.33	0.33	0.33	0.50	0.30	0.20	0.50	0.40	0.10	0.50	0.45	0.05	0.46	0.45	0.09	0.80	0.10	0.10
g=4	0.33	0.33	0.33	0.50	0.30	0.20	0.50	0.40	0.10	0.50	0.45	0.05	0.46	0.45	0.09	0.80	0.10	0.10
g=1	0.80	0.15	0.05	0.80	0.19	0.01	0.90	0.05	0.05	0.90	0.08	0.02	0.90	0.09	0.01	0.20	0.30	0.50
g=2	0.80	0.15	0.05	0.80	0.19	0.01	0.90	0.05	0.05	0.90	0.08	0.02	0.90	0.09	0.01	0.20	0.30	0.50
g=3	0.80	0.15	0.05	0.80	0.19	0.01	0.90	0.05	0.05	0.90	0.08	0.02	0.90	0.09	0.01	0.20	0.30	0.50
g=4	0.80	0.15	0.05	0.80	0.19	0.01	0.90	0.05	0.05	0.90	0.08	0.02	0.90	0.09	0.01	0.20	0.30	0.50
g=1	0.10	0.40	0.50	0.05	0.45	0.50	0.08	0.45	0.46	0.10	0.10	0.80	0.05	0.15	0.80	0.01	0.19	0.80
g=2	0.10	0.40	0.50	0.05	0.45	0.50	0.08	0.45	0.46	0.10	0.10	0.80	0.05	0.15	0.80	0.01	0.19	0.80
g=3	0.10	0.40	0.50	0.05	0.45	0.50	0.08	0.45	0.46	0.10	0.10	0.80	0.05	0.15	0.80	0.01	0.19	0.80
g=4	0.10	0.40	0.50	0.05	0.45	0.50	0.08	0.45	0.46	0.10	0.10	0.80	0.05	0.15	0.80	0.01	0.19	0.80
g=1	0.05	0.05	0.90	0.02	0.08	0.90	0.01	0.09	0.90	0.33	0.33	0.33	0.50	0.30	0.20	0.50	0.40	0.10
g=2	0.05	0.05	0.90	0.02	0.08	0.90	0.01	0.09	0.90	0.33	0.33	0.33	0.50	0.30	0.20	0.50	0.40	0.10
g=3	0.05	0.05	0.90	0.02	0.08	0.90	0.01	0.09	0.90	0.50	0.30	0.20	0.50	0.40	0.10	0.50	0.45	0.05
g=4	0.05	0.05	0.90	0.02	0.08	0.90	0.01	0.09	0.90	0.50	0.30	0.20	0.50	0.40	0.10	0.50	0.45	0.05
g=1	0.50	0.45	0.05	0.46	0.45	0.09	0.80	0.10	0.10	0.80	0.15	0.05	0.80	0.19	0.01	0.90	0.05	0.05
g=2	0.50	0.45	0.05	0.46	0.45	0.09	0.80	0.10	0.10	0.80	0.15	0.05	0.80	0.19	0.01	0.90	0.05	0.05
g=3	0.46	0.45	0.09	0.80	0.10	0.10	0.80	0.15	0.05	0.80	0.19	0.01	0.80	0.15	0.05	0.90	0.08	0.02
g=4	0.80	0.10	0.10	0.80	0.15	0.05	0.80	0.19	0.01	0.90	0.05	0.05	0.80	0.10	0.10	0.90	0.09	0.01
g=1	0.90	0.08	0.02	0.20	0.30	0.50	0.10	0.40	0.50	0.05	0.45	0.50	0.08	0.45	0.47	0.10	0.10	0.80
g=2	0.90	0.09	0.01	0.20	0.30	0.50	0.10	0.40	0.50	0.05	0.45	0.50	0.08	0.45	0.47	0.10	0.10	0.80
g=3	0.90	0.09	0.01	0.33	0.33	0.33	0.20	0.30	0.50	0.10	0.40	0.50	0.05	0.45	0.50	0.05	0.45	0.50
g=4	0.90	0.09	0.01	0.33	0.33	0.33	0.33	0.33	0.33	0.20	0.30	0.50	0.08	0.45	0.47	0.10	0.40	0.50
g=1	0.05	0.15	0.80	0.01	0.19	0.80	0.05	0.05	0.90	0.02	0.08	0.90	0.33	0.33	0.33	0.50	0.30	0.20
g=2	0.05	0.15	0.80	0.01	0.19	0.80	0.05	0.05	0.90	0.01	0.09	0.90	0.33	0.33	0.33	0.50	0.30	0.20
g=3	0.10	0.10	0.80	0.05	0.15	0.80	0.05	0.15	0.80	0.01	0.09	0.90	0.20	0.30	0.50	0.33	0.33	0.33
g=4	0.08	0.45	0.47	0.10	0.10	0.80	0.08	0.45	0.46	0.01	0.09	0.90	0.20	0.30	0.50	0.20	0.30	0.50
g=1	0.50	0.40	0.10	0.50	0.45	0.05	0.46	0.45	0.09	0.80	0.10	0.10	0.80	0.15	0.05	0.80	0.19	0.01
g=2	0.50	0.40	0.10	0.50	0.40	0.10	0.46	0.45	0.09	0.80	0.10	0.10	0.80	0.15	0.05	0.80	0.19	0.01
g=3	0.50	0.30	0.20	0.50	0.45	0.05	0.50	0.45	0.05	0.46	0.45	0.09	0.80	0.10	0.10	0.46	0.45	0.09
g=4	0.33	0.33	0.33	0.20	0.30	0.50	0.20	0.30	0.50	0.10	0.40	0.50	0.46	0.45	0.09	0.05	0.45	0.50
g=1	0.90	0.05	0.05	0.90	0.08	0.02	0.20	0.30	0.50	0.10	0.40	0.50	0.05	0.45	0.50	0.08	0.45	0.46
g=2	0.90	0.05	0.05	0.90	0.05	0.05	0.20	0.30	0.50	0.10	0.40	0.50	0.05	0.45	0.50	0.08	0.45	0.46
g=3	0.80	0.19	0.01	0.80	0.19	0.01	0.10	0.40	0.50	0.05	0.45	0.50	0.08	0.45	0.47	0.10	0.10	0.80
g=4	0.20	0.30	0.05	0.80	0.15	0.05	0.05	0.45	0.50	0.08	0.45	0.47	0.10	0.10	0.80	0.05	0.15	0.80
g=1	0.10	0.10	0.80	0.05	0.15	0.80	0.01	0.19	0.80	0.05	0.05	0.90	0.02	0.08	0.90	0.90	0.05	0.05
g=2	0.10	0.10	0.80	0.05	0.15	0.80	0.01	0.19	0.80	0.05	0.05	0.90	0.05	0.05	0.90	0.05	0.90	0.05
g=3	0.05	0.15	0.80	0.01	0.19	0.80	0.05	0.05	0.90	0.02	0.08	0.90	0.01	0.19	0.80	0.05	0.05	0.90
g=4	0.01	0.19	0.80	0.05	0.05	0.90	0.02	0.08	0.90	0.01	0.09	0.90	0.05	0.15	0.80	0.33	0.33	0.33
