# Supplementary material for: Multiple Comparisons With Overdispersed Multinomial Data: Methods, Properties and Application
Source: Pharm Stat. 2026 Jan 19;25(1):e70073. doi: 10.1002/pst.70073 (PMC12815589; doi:10.1002/pst.70073)
Supplement: Supplementary file 1 — Data S1: pst70073‐sup‐0001‐supinfo.pdf. [file PST-25-0-s001.pdf]

Supplementary Material for:
Multiple Comparisons with Overdispersed Multinomial Data: Methods, Properties and Application

Sören Budig[1], Charlotte Vogel[2], and Frank Schaarschmidt[1,*]
[1]Department of Biostatistics, Leibniz University Hannover, Hannover, Germany
[2]Independent Researcher, Germany
[*]Corresponding author: budig@cell.uni-hannover.de

November 26, 2025

Abstract

This document provides supplementary information for the manuscript titled "Multiple Comparisons with Overdispersed Multinomial Data: Methods, Properties and Application". It includes additional simulation studies designed to evaluate the robustness of the methods presented in the manuscript under various conditions. All items are numbered with an 'S' prefix.

Contents

S1 Additional Simulation Studies **3**
S1.1 Variable Cluster Sizes 3
S1.1.1 Fixed α Parameter Across Clusters 3
S1.1.2 Cluster-Specific α Parameter 3
S1.1.3 Simulation Setup 3
S1.1.4 Results 3
S1.2 Heterogenous Dispersion Across Groups 5
S1.3 Logistic Normal Multinomial 5

S2 Computational Performance and Scalability **8**

S1 Additional Simulation Studies

S1.1 Variable Cluster Sizes

The main simulation was conducted under the simplifying assumption that all clusters within an experiment have the same size ($m_{gb} = m$). While this is a straightforward approach in simulation studies, many real-world datasets, particularly in fields like toxicology and ecology, exhibit variability in cluster sizes. For instance, the number of offspring per litter can vary, or the number of individuals captured in a sample might not be constant. This variability could potentially affect the performance of the statistical methods examined, especially the estimation of the dispersion parameter. Therefore, we conducted two additional simulation studies to assess the impact of variable cluster sizes. In both subsequent approaches, the cluster size m_{gb} is drawn from a Poisson distribution.

S1.1.1 Fixed α Parameter Across Clusters

In the first approach, we simulated a scenario in which the parameterisation of the Dirichlet distribution remained constant across clusters, regardless of their size. To achieve this, we first calculated a single Dirichlet shape parameter, α, based on the expected (average) cluster size, m, and the target dispersion, ϕ, using the formula $\alpha = (m - \phi)/(\phi - 1)$. This fixed α was then used to generate a unique probability vector, $\boldsymbol{\pi}_{gb}$, for each cluster by sampling from a Dirichlet distribution with parameters $\alpha\boldsymbol{\pi}_g$. The actual size for each cluster, m_{gb}, was then drawn independently from a Poisson distribution with mean m.

S1.1.2 Cluster-Specific α Parameter

In the second approach, we explored a scenario where overdispersion is linked to individual cluster size. Here, the cluster sizes, m_{gb}, were first drawn from a Poisson distribution with mean m. Then, for each cluster, a specific Dirichlet shape parameter, α_{gb}, was calculated using its individual size m_{gb} and the target dispersion ϕ: $\alpha_{gb} = (m_{gb} - \phi)/(\phi - 1)$. This cluster-specific α_{gb} was then used to generate the probability vector $\boldsymbol{\pi}_{gb}$ for that cluster. This approach creates a situation in which the degree of overdispersion, as expressed by the parameter ϕ, remains fixed despite the variation in m_{gb}.

S1.1.3 Simulation Setup

For both simulation setups, we used a subset of 20 scenarios for the true proportions from the main text (corresponding to the first and third columns of Table A1 in the main manuscript). Average cluster sizes were $m \in \{10, 20, 50, 100\}$, with $B_g \in \{5, 10, 20, 50\}$ clusters per group. Since individual cluster sizes m_{gb} were drawn from a Poisson(m) distribution, the 2.5% and 97.5% quantiles (capturing the central 95% of the distribution) for the cluster sizes used were approximately 4–17 ($m = 10$), 12–29 ($m = 20$), 37–64 ($m = 50$), and 81–120 ($m = 100$). The overdispersion parameter was $\phi \in \{1.01, 2, 5\}$. Datasets were analysed using the methods from the main text, restricted to the MGLM implementation for the DM model and Tukey-type contrasts, consistent with other supplementary simulations.

S1.1.4 Results

By evaluating the methods under these conditions, we aimed to determine if the quasi-likelihood estimators can still maintain robust control of the Family-Wise Error Rate (FWER) when cluster sizes are varied. Figure S1 (fixed α) and Figure S2 (cluster-specific α) present the FWER results. Both approaches yield very similar outcomes, consistent with the main simulation findings. The Pearson, Farrington, and Afroz quasi-likelihood methods control the FWER across

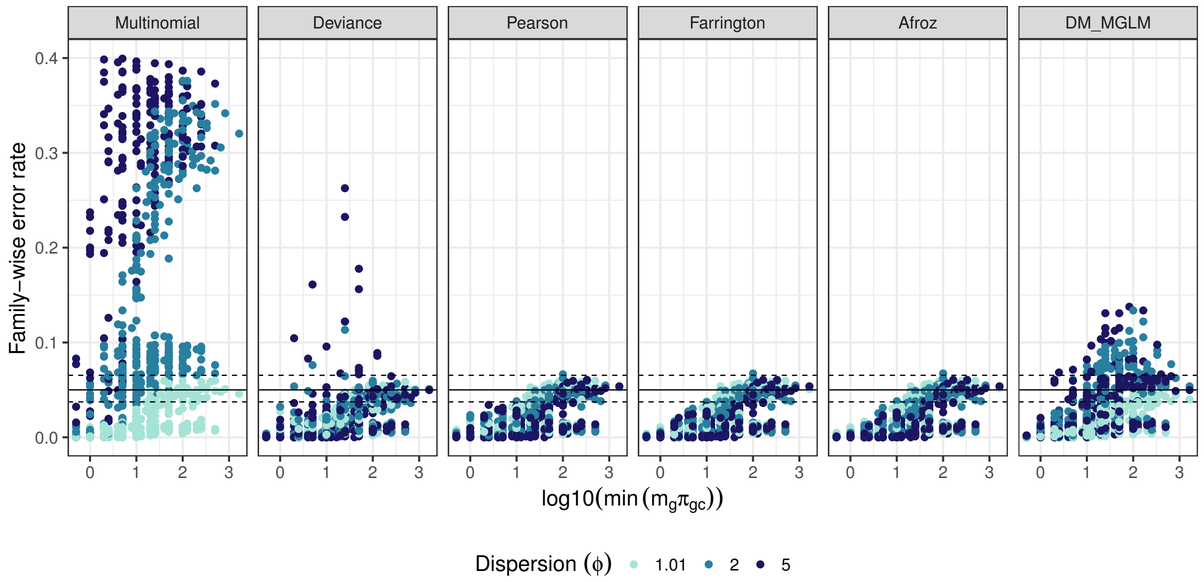

Figure S1: Simulated FWER with variable cluster sizes and a fixed α parameter. Colour indicates the target dispersion ϕ. The nominal 0.05 level (solid line) and simulation standard error bounds (dashed lines) are shown. The y-axis limit is 0.4.

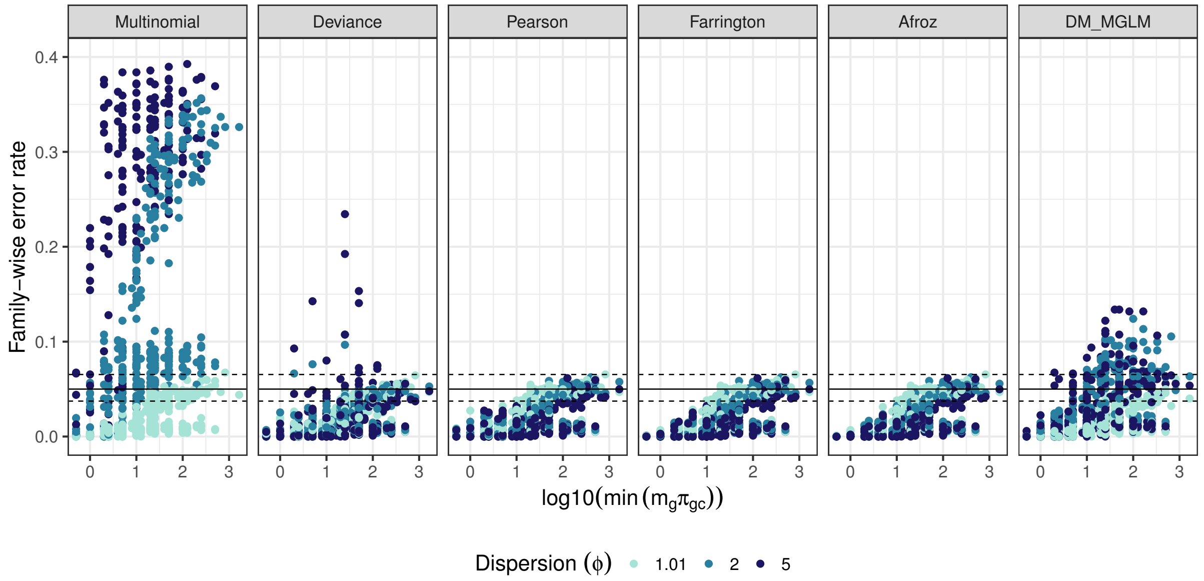

Figure S2: Simulated FWER with variable cluster sizes and cluster-specific α parameters. Colour indicates the target dispersion ϕ. The nominal 0.05 level (solid line) and simulation standard error bounds (dashed lines) are shown. The y-axis limit is 0.4.

all tested scenarios. In contrast, the Dirichlet-multinomial model (MGLM implementation) exhibits slightly liberal behaviour (FWER up to 0.14) in some cases, particularly at higher dispersion levels, mirroring the results from the main text. Variable cluster sizes, under these data generation schemes, did not substantially alter the FWER control properties compared to the equal cluster size scenario.

S1.2 Heterogenous Dispersion Across Groups

The main simulation study assumed homogeneous dispersion (i.e., a consistent level of overdispersion across all treatment groups). However, in real-world applications, it is plausible that different treatments could induce different levels of variability in the multinomial counts. For example, a high dose of a toxic substance might not only shift the mean proportions of outcomes but also increase the variability among subjects within that group compared to a control group. To investigate the robustness of the proposed methods to heterogeneous, or group-specific, dispersion, we conducted a simulation study.

The data generation process used the Dirichlet-multinomial distribution but was adapted for varying overdispersion levels. Instead of a single ϕ, we used a vector $\boldsymbol{\phi} = (\phi_1, \phi_2, \phi_3, \phi_4)$, assigning a specific dispersion parameter ϕ_g to each treatment group g. Data for each group were generated independently using its assigned ϕ_g.

We used 20 proportion scenarios (first and third columns of Table A1 in the main manuscript). The number of clusters was $B_g \in \{5, 10, 20, 50\}$, and cluster sizes were $m_{gb} \in \{10, 20, 50, 100\}$. We focused on five patterns of heterogeneity using different $\boldsymbol{\phi}$ vectors:

- One group with no overdispersion and three with high overdispersion: $(1.01, 5, 5, 5)$
- A gradient of increasing overdispersion: $(1.01, 2, 5, 8)$
- A mix of low and very high overdispersion: $(1.01, 1.5, 8, 1.5)$ and $(1.01, 8, 1.01, 8)$
- All groups having medium to high overdispersion: $(1.5, 1.5, 5, 8)$

Generated datasets were analysed using the same methods as in the simulation before (MGLM implementation for DM, Tukey contrasts for groups). Since quasi-likelihood approaches estimate a single, global dispersion parameter, this simulation tests their performance when averaging over different true dispersion levels. The question was whether this global estimate is sufficient to maintain control of the FWER when the underlying assumption of homogeneous dispersion is not met. Figure S3 shows the FWER under heterogeneous dispersion. All methods exhibit liberal behaviour in some scenarios. The Pearson, Farrington, and Afroz quasi-likelihood estimators perform similarly, with FWER reaching up to 0.1, particularly when dispersion levels varied widely (e.g., mixing low and very high ϕ). However, FWER control was maintained in many other settings. The Dirichlet-multinomial model also controlled FWER in many cases but sometimes reached 0.125, seemingly less dependent on the specific dispersion pattern compared to quasi-likelihood methods. Overall, heterogeneity can inflate the FWER, especially for quasi-likelihood methods when dispersion levels are substantially different across groups.

S1.3 Logistic Normal Multinomial

The main simulation used the Dirichlet-multinomial (DM) distribution for data generation. To assess robustness to model misspecification, we conducted simulations using the logistic normal multinomial (LNM) model, which allows for a more flexible covariance structure, including positive correlations between category proportions, unlike the DM model.

The LNM data-generating process was carried out in three main steps for each group g. First, we defined the mean vector $\boldsymbol{\mu}_g$ for a set of latent variables by applying an additive log-ratio transformation to the true proportions $\boldsymbol{\pi}_g$. Using the last category (C) as the reference,

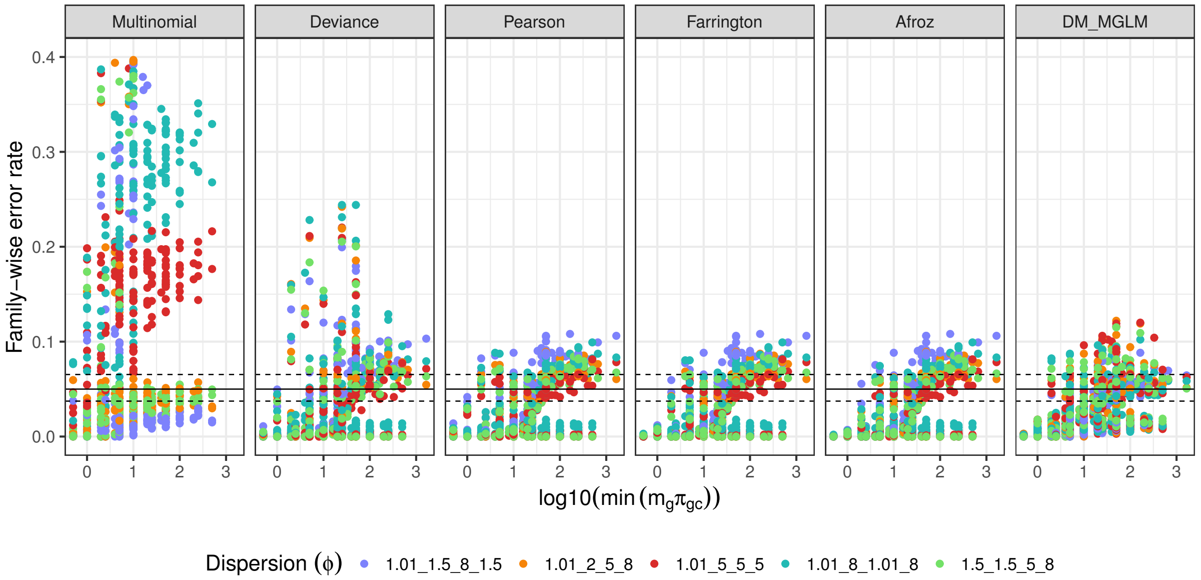

Figure S3: Simulated FWER with heterogeneous dispersion across groups. Colour indicates the specific combination of group dispersions ($\phi_1, \phi_2, \phi_3, \phi_4$). The nominal 0.05 level (solid line) and simulation standard error bounds (dashed lines) are shown. The y-axis limit is 0.4.

the elements of $\boldsymbol{\mu}_g$ were computed as $\mu_{gc} = \log(\pi_{gc}/\pi_{gC})$ for $c = 1, \ldots, C-1$. Second, for each of the B_g clusters, we sampled a $(C-1)$-dimensional vector of latent variables, $\boldsymbol{z}_{gb}$, from a multivariate normal (MVN) distribution, $\boldsymbol{z}_{gb} \sim \text{MVN}(\boldsymbol{\mu}_g, \boldsymbol{\Sigma})$, where $\boldsymbol{\Sigma}$ is a specified covariance matrix that controls the level of overdispersion and correlation between categories. The variances (the diagonal elements of $\boldsymbol{\Sigma}$) control the magnitude of the overdispersion. Larger variances result in greater variability among the sampled latent vectors $\boldsymbol{z}_{gb}$. When these are back-transformed, they produce more heterogeneous probability vectors $\boldsymbol{p}_{gb}$ across clusters, leading to greater variability in the final counts than expected under a simple multinomial model. The covariances (the off-diagonal elements of $\boldsymbol{\Sigma}$) determine the correlation structure between the category counts. Unlike the Dirichlet-multinomial model, which only allows for negative correlations between proportions, the LNM model can capture both positive and negative correlations through the covariance terms.

Third, these latent variables were back-transformed into a unique probability vector $\boldsymbol{\pi}_{gb}$ for each cluster using:

$$\pi_{gbc} = \frac{\exp(z_{gbc})}{1 + \sum_{j=1}^{C-1} \exp(z_{gbj})} \quad \text{for } c = 1, \ldots, C-1,$$

where j indexes the $C-1$ latent variables corresponding to the non-reference categories. The probability for the reference category is determined by $\pi_{gbC} = 1 - \sum_{c=1}^{C-1} \pi_{gbc}$. Finally, the observed count vector $\boldsymbol{y}_{gb}$ for each cluster was generated by sampling from a multinomial distribution with size m_{gb} and the cluster-specific probability vector $\boldsymbol{\pi}_{gb}$, i.e., $\boldsymbol{y}_{gb} \sim \text{Multinomial}(m_{gb}, \boldsymbol{\pi}_{gb})$.

We used 20 proportion scenarios (first and third columns of Table A1 in the main manuscript). Cluster numbers ($B_g \in \{5, 10, 20, 50\}$) and sizes ($m_{gb} \in \{10, 20, 50, 100\}$) matched the main simulation. The overdispersion was controlled by specifying the elements of the covariance matrix $\boldsymbol{\Sigma}$ (dimension 2×2 for $C = 3$). We varied the diagonal elements (variances) with values in $\{0.1, 1, 5\}$ and set the off-diagonal elements (covariances) with values in $\{0, 0.5, 1\}$, considering only combinations where the covariance was less than or equal to the variance.

It should be noted that a variance of 5 on the logit scale is considered quite extreme. This implies a standard deviation of approximately 2.24. Since the 95% range of a normal distribution spans roughly $\pm 2\sigma$ (a width of ≈ 9 on the logit scale), and the logit transformation of

probabilities from 0.01 to 0.99 spans a range of approximately 9.2, this level of variance implies that cluster-specific probabilities can fluctuate across almost the entire $(0,1)$ interval.

This resulted in the following covariance matrices used for the simulation:

$$\begin{pmatrix} 0.1 & 0 \\ 0 & 0.1 \end{pmatrix}, \begin{pmatrix} 1 & 0 \\ 0 & 1 \end{pmatrix}, \begin{pmatrix} 1 & 0.5 \\ 0.5 & 1 \end{pmatrix}, \begin{pmatrix} 1 & 1 \\ 1 & 1 \end{pmatrix}, \begin{pmatrix} 5 & 0 \\ 0 & 5 \end{pmatrix}, \begin{pmatrix} 5 & 0.5 \\ 0.5 & 5 \end{pmatrix}, \begin{pmatrix} 5 & 1 \\ 1 & 5 \end{pmatrix}$$

We evaluated quasi-likelihood estimators and the MGLM implementation of the DM model using Tukey contrasts. The goal was assessing FWER control under this model misspecification (analysing LNM data with methods assuming DM or quasi-multinomial structures).

Furthermore, to assess the combined impact of model misspecification and variable cluster sizes, we conducted an additional simulation. This simulation used the identical LNM data-generating process, covariance matrix parameters ($\mathbf{\Sigma}$), proportion scenarios, and methods as described above. However, instead of a fixed cluster size m_{gb}, the size for each individual cluster was drawn from a Poisson distribution with a mean $m \in \{10, 20, 50, 100\}$.

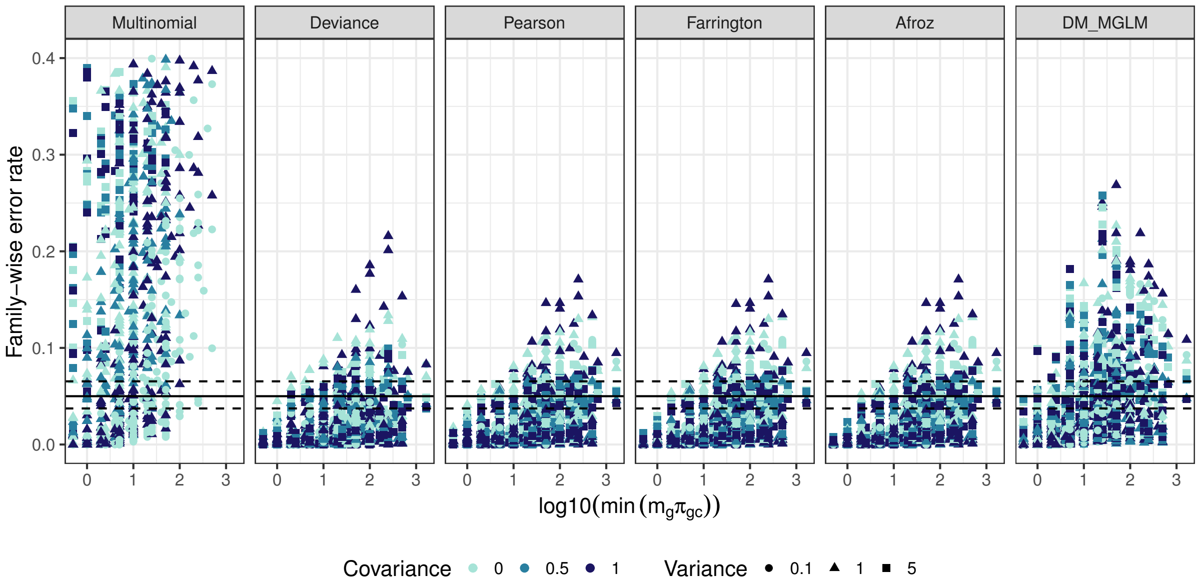

Figure S4: Simulated FWER using the LNM data generating process with fixed cluster sizes. Colour indicates the covariance level, shape indicates the variance level. The nominal 0.05 level (solid line) and simulation standard error bounds (dashed lines) are shown. The y-axis limit is 0.4.

Figure S4 shows the FWER when analysing LNM-generated data with fixed cluster sizes. Many settings result in liberal behaviour for all methods. Quasi-likelihood methods reach FWER up to 0.2, while the DM model reaches 0.38. Liberal behaviour is particularly pronounced when the covariance and variance are both high. For quasi-likelihood methods, zero covariance sometimes leads to higher FWER than moderate covariance (0.5). Generally, higher variance increases FWER under this misspecification.

Figure S5 displays FWER for LNM data with variable cluster sizes. While the general pattern of results closely mirrors that of the fixed cluster size case (Figure S4), slight differences are observable. Specifically, in certain settings, the introduction of variable cluster sizes results in a marginal additional inflation of the FWER. This suggests that while the mismatch between the LNM's flexible correlation structure and the assumptions of the multinomial or DM model remains the primary driver of the inflated error rates, the additional variability introduced by unequal cluster sizes can further compromise error control in specific scenarios.

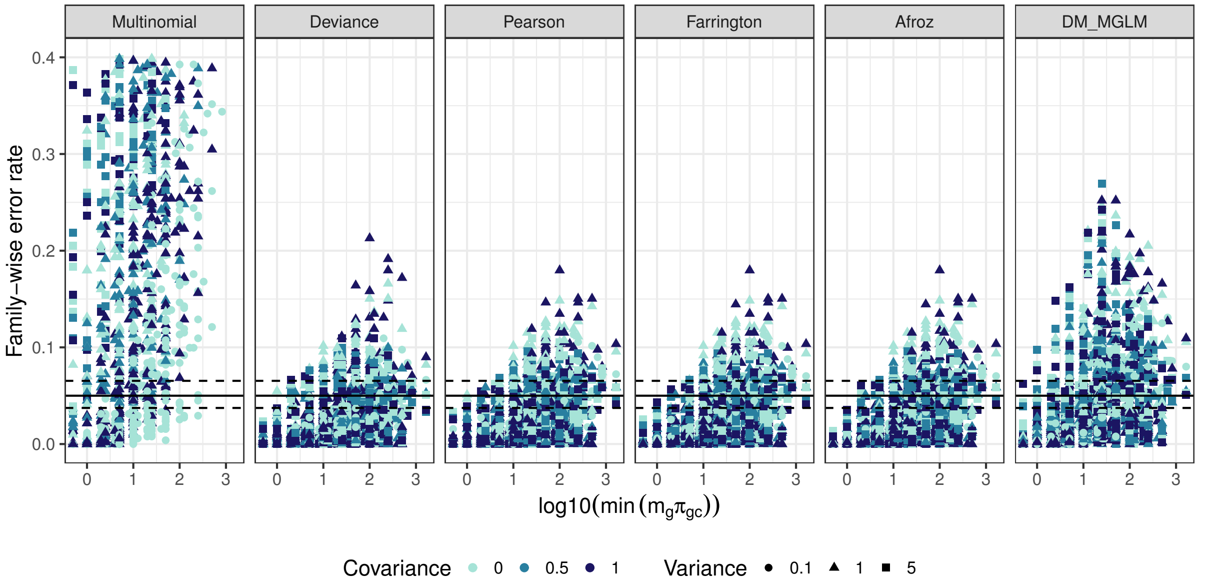

Figure S5: Simulated FWER using the LNM data generating process with variable cluster sizes. Colour indicates the covariance level, shape indicates the variance level. The nominal 0.05 level (solid line) and simulation standard error bounds (dashed lines) are shown. The y-axis limit is 0.4.

S2 Computational Performance and Scalability

We conducted a simulation study to evaluate the computational cost and scalability of the proposed methods. The practical feasibility of these methods depends on the computational time required for both model fitting and the subsequent multiple comparison adjustment. This study was designed to assess these two components separately as the number of groups (G) and categories (C) increases, as both factors drive the total number of comparisons.

We measured the computation time (in seconds) for three model-fitting approaches: the multinomial quasi-likelihood approach fit via `vglm` (used for the Afroz estimator), the Dirichlet-multinomial (DM) model implemented via `VGAM`, and the DM model implemented via `MGLM`.

Simulations were run on a full grid of parameters, varying the number of groups $G \in \{3, 5, 10, 15, 20\}$ and the number of categories $C \in \{3, 5, 10, 15, 20\}$. We also included three cluster sizes $m \in \{10, 20, 50\}$. Other parameters were held constant: the number of clusters per group ($b = 10$) and the overdispersion ($\phi = 1.5$). Tukey-type (all-pairs) contrasts were computed between groups, with the first category as the logit baseline. This results in $M = (C - 1) \times [G(G - 1)/2]$ total simultaneous comparisons.

For each combination of parameters (G, C, m, and model type), 20 replicates were run. We recorded the average computation time for two distinct steps: the model fitting time and the multiple comparison adjustment time. The adjustment step involves the numerical integration of a multivariate t-distribution, and its difficulty scales with the number of comparisons. All simulations were performed using `R` software version 4.3.3 on an AMD Ryzen 9 9950X CPU (approximately 5.7 GHz per core).

Figure S6 shows the average computation time for model fitting. The QL model (fit via `vglm`) was consistently the fastest, with average times ranging from 0.006 seconds (C=3, G=3, m=50) to 3.88 seconds (C=20, G=20, m=10). In contrast, the `VGAM` implementation of the DM model was frequently the slowest, with times ranging from 0.007 seconds (C=3, G=5, m=20) to 1197 seconds (C=20, G=20, m=50). The `MGLM` implementation was often as fast as the QL model for smaller scenarios, but its computation time increased with the number of categories, groups, and cluster sizes, even exceeding the `VGAM` implementation in the largest setting (1655

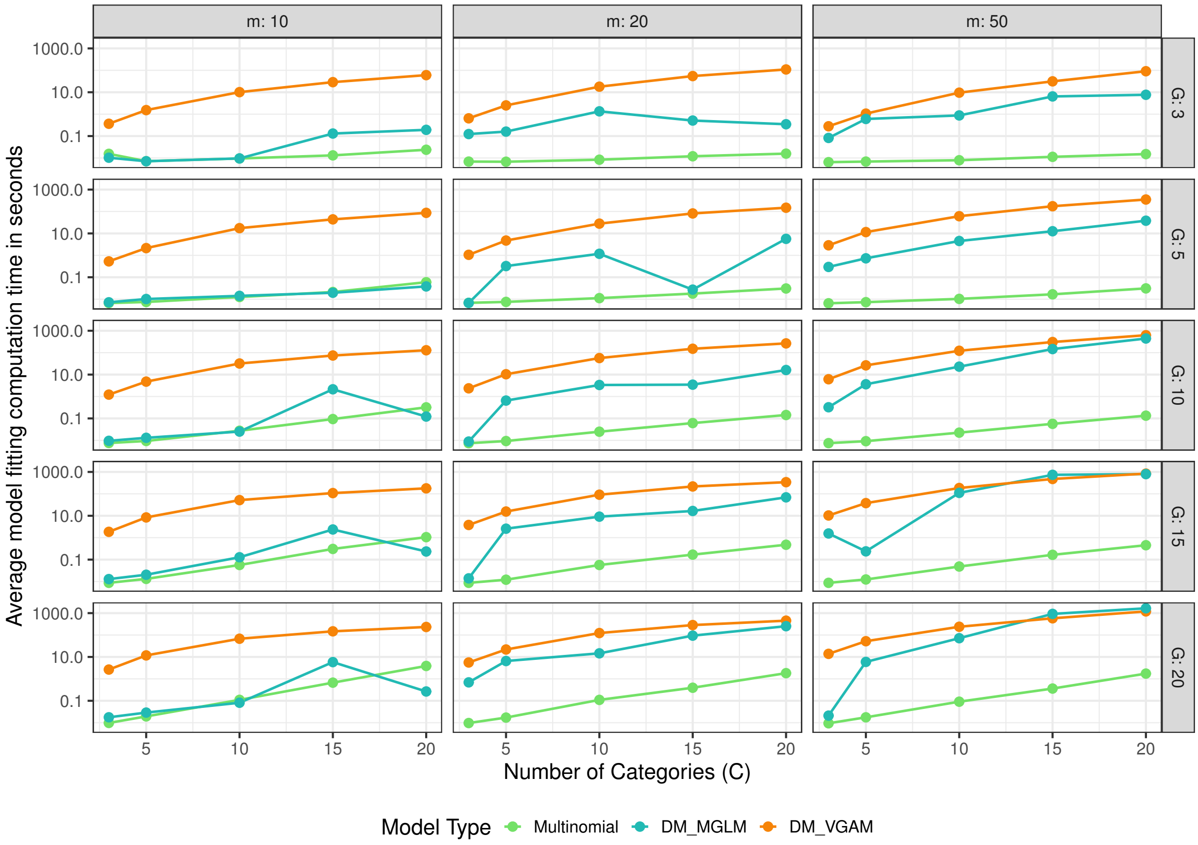

Figure S6: Average computation time (seconds, log10 scale) for model fitting for the three model types. The number of categories (C) are displayed on the x-axis, the number of groups (G) on the row facets. The cluster sizes (m) are represented by the column facets.

seconds for C=20, G=20, m=50).

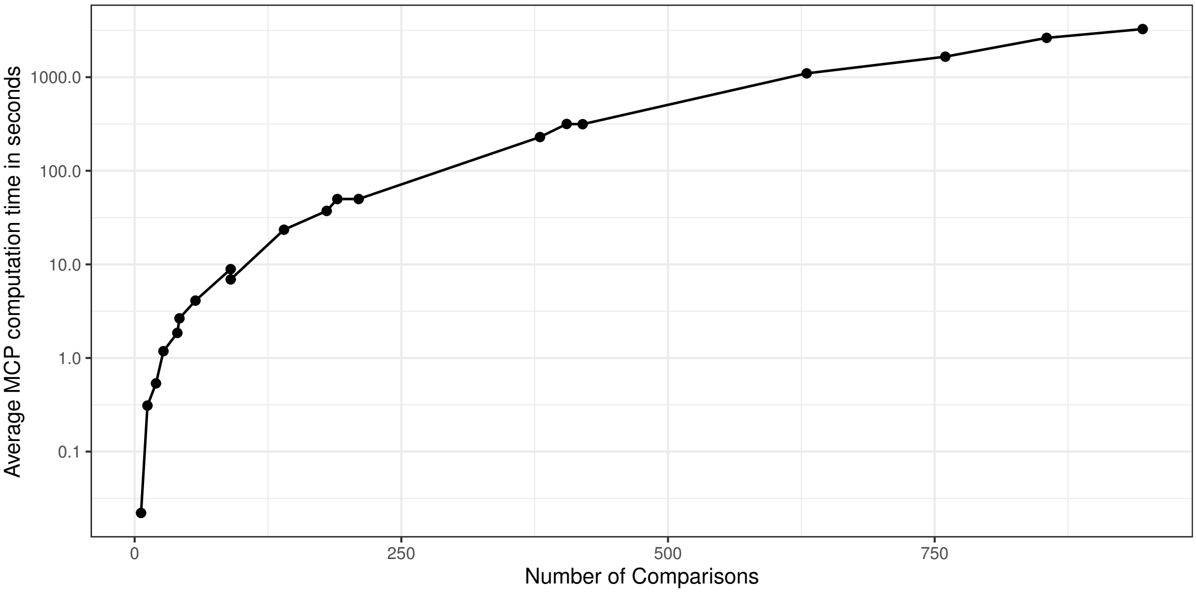

Figure S7: Average computation time (seconds, log10 scale) for the multiple comparison procedure as a function of the number of comparisons. Results are averaged across cluster sizes ($m \in \{10, 20, 50\}$) and model types.

Figure S7 shows the average computation time for the multiple comparison procedure. Results were averaged over all replicates, model types, and cluster sizes, as these factors did not substantially influence the adjustment time. The `pmvt` function, which computes the adjusted p-values, has a practical limit of 1000 comparisons. Therefore, the setting with the highest number of comparisons shown is C=10 and G=15 (945 comparisons). It is clear that the average computation time increases rapdily with the number of comparisons. The largest scenario shown took an average of 3265 seconds.

In summary, all methods are computationally feasible for typical study sizes. For the QL approaches, the primary driver of computational cost is the multiplicity adjustment, which becomes intensive as the total number of comparisons grows. For the DM models, both the model fitting and the adjustment step can be computationally demanding in large-scale scenarios.
